# Angiotensin-Converting Enzyme (ACE) Inhibitors and Angiotensin Receptor Blockers (ARB) Are Protective Against ICU Admission and Mortality for Patients With COVID-19 Disease

**DOI:** 10.3389/fmed.2021.600385

**Published:** 2021-03-04

**Authors:** Rawan ElAbd, Dana AlTarrah, Sarah AlYouha, Hamad Bastaki, Sulaiman Almazeedi, Mohannad Al-Haddad, Mohammad Jamal, Salman AlSabah

**Affiliations:** ^1^Faculty of Medicine, Kuwait University, Kuwait City, Kuwait; ^2^Faculty of Public Health, Kuwait University, Safat, Kuwait; ^3^Jaber Al Ahmed Al Sabah Health Center, Ministry of Health (Kuwait), Kuwait City, Kuwait; ^4^Public Health Department, Ministry of Health, Kuwait City, Kuwait

**Keywords:** ACEi (angiotensin conversing enzyme inhibitor), ARB (angiotensin II AT1 receptor blocker), ICU–intensive care unit, COVID−19, mortality

## Abstract

**Introduction:** Corona Virus disease 2019 (COVID-19) caused by the Severe Acute Respiratory Syndrome coronavirus 2 (SARS-CoV-2) has become a global pandemic. The aim of this study was to investigate the impact of being on an Angiotensin-Converting Enzyme Inhibitors (ACEI) and/or Angiotensin Receptor Blockers (ARB) on hospital admission, on the following COVID-19 outcomes: disease severity, ICU admission, and mortality.

**Methods:** The charts of all patients consecutively diagnosed with COVID-19 from the 24th of February to the 16th of June of the year 2020 in Jaber Al-Ahmed Al-Sabah hospital in Kuwait were checked. All related patient information and clinical data was retrieved from the hospitals electronic medical record system. The primary outcome was COVID-19 disease severity defined as the need for Intensive Care Unit (ICU) admission. Secondary outcome was mortality.

**Results:** A total of 4,019 COVID-19 patients were included, of which 325 patients (8.1%) used ACEI/ARB, users of ACEI/ARB were found to be significantly older (54.4 vs. 40.5 years). ACEI/ARB users were found to have more co-morbidities; diabetes (45.8 vs. 14.8%) and hypertension (92.9 vs. 13.0%). ACEI/ARB use was found to be significantly associated with greater risk of ICU admission in the unadjusted analysis [OR, 1.51 (95% CI: 1.04–2.19), *p* = 0.028]. After adjustment for age, gender, nationality, coronary artery disease, diabetes and hypertension, ICU admission was found to be inversely associated with ACEI use [OR, 0.57 (95% CI: 0.34–0.88), *p* = 0.01] and inversely associated with mortality [OR, 0.56 (95% CI: 0.33–0.95), *p* = 0.032].

**Conclusion:** The current evidence in the literature supports continuation of ACEI/ARB medications for patients with co-morbidities that acquire COVID-19 infection. Although, the protective effects of such medications on COVID-19 disease severity and mortality remain unclear, the findings of the present study support the use of ACEI/ARB medication.

## Introduction

Corona Virus disease 2019 (COVID-19) caused by the Severe Acute Respiratory Syndrome coronavirus 2 (SARS-CoV-2) has become a global pandemic (1). Although the disease is easily transmissible, clinical presentation ranges from being asymptomatic to multi-organ involvement and death (2–5). Disease severity has been found to be associated with certain risk factors like older age, male gender, and co-morbidities (6, 7). The overall mortality rate has been found to range from 1 to 5% (6). However, in the presence of cardiovascular disease, diabetes, chronic respiratory disease, or hypertension, the mortality rate is found to increase dramatically (8, 9).

SARS-CoV-2 enters human cell through the angiotensin-converting enzyme 2 (ACE2) receptor, a membrane receptor that is broadly expressed in the respiratory system, the gastrointestinal tracts, the heart, and the kidney (10–12). Due to its close association with the ACE2 receptor, concerns were raised about the effect of using antihypertensive medications like angiotensin-converting enzyme inhibitors (ACEIs) or angiotensin receptor blockers (ARBs) in patients with COVID-19. It has been hypothesized that the use of such drugs could upregulate the ACE2 receptor expression in alveolar 2 cells (13) and render patients more susceptible to infection and disease propagation. On the other hand, it was suggested that the use of such drugs may inhibit the ACE2 receptor and prevent virus entry to the cell, thus posing a protective effect (14). The aim of this study was to investigate the impact of being on an Angiotensin-Converting Enzyme Inhibitors (ACEI) and/or Angiotensin Receptor Blockers (ARB) on hospital admission, on the following COVID-19 outcomes: disease severity, ICU admission, and mortality.

## Methods

### Study Design and Data Collection

For the present retrospective study, all patients consecutively diagnosed with COVID-19 from the 24th of February to the 16th of June of the year 2020 in Jaber Al-Ahmed Al-Sabah hospital in Kuwait were included in the study. Inclusion criteria included patients of all ages diagnosed with COVID-19 using PCR testing, in accordance with the World Health Organization (WHO) interim guidance (15). All related patient information and clinical data was retrieved from the hospitals electronic medical record system. These included sociodemographic factors (age, gender, nationality), clinical indicators (temperature on admission, blood pressure), and presence of co-morbidities (diabetes, hypertension, asthma, coronary artery disease).

#### Laboratory Investigations

All diagnostic tests were performed in Jaber Al-Ahmad Al-Sabah hospital in Kuwait. COVID-19 was confirmed via real-time reverse-transcriptase-polymerase chain-reaction (RT-PCR) assay of specimens obtained via nasopharyngeal swabs (16).

### Outcome

For the present study the two investigated outcomes were compared across ACEI/ARB and non-ACEI/ARB users. The primary outcome was COVID-19 disease severity measured as the need for Intensive Care Unit (ICU) admission. The secondary outcome was mortality. All patient mortalities were attributed to COVID-19 since only SARS-CoV-2 positive patients were admitted and subsequently included in the present study. Criteria for ICU admission was based upon patients need for mechanical ventilation and/or vasopressors which was determined based upon evaluation by a rapid response COVID-19 team who assess individual patients with certain risk factors: age > 60 years old, heart rate > 100, systolic blood pressure <90 or mean arterial pressure <65, temperature >38.1, respiratory rate >26–30, saturation of oxygen <92% on room air, or any pulmonary infiltrate not considered chronic changes. The presence of any 3 of the previous criteria alerts the COVID-19 team to discuss with the ICU consultant on-call for decision regarding ICU admission. Our center's ICU beds were never fully occupied, and any patient with the above stated indications was admitted to ICU.

Patients who were ACE/ARB users on hospital admission, were compared to those who were not on those medications when they first presented to the hospital.

### Ethical Considerations and Role of Funding

Ethical approval for conduction of this study was granted by the Ministry of Health Ethical Review Board in Kuwait (No. 2020/1402). A research grant (Grant No.: Cor-prop-35) was awarded by the Kuwait Foundation for the Advancement of Science (KFAS) and was utilized for assistance in data collection, statistical analysis, and publication.

### Statistical Analysis

Data was analyzed using R (version 4) (17). Descriptive statistics were used to report mean and standard deviations for continuous data, and frequency statistics were used to calculate numbers and percentages for categorical variables.

Patient characteristics of ACE/ARB and non-ACE/ARB users were analyzed using independent t-test for continuous variables and, chi-squared test for categorical variables.

Logistic regression was used to identify significant predictors of ACE and non-ACE users, in both unadjusted and adjusted models. Unadjusted models were first run separately for each factor, followed by multiple logistic regression models. Models were adjusted for the following covariates: age, gender, non-Kuwaiti, CAD, diabetes and hypertension. Odds Ratio (OR) with 95% Confidence Intervals (CI) was calculated. Statistical significance was set at *p* value < 5%.

## Results

In the present retrospective cohort study 4,019 COVID-19 patients were included, of which 325 patients (8.1%) used ACEI/ARB, whilst 3,694 (91.9%) did not. Baseline patient characteristics are shown in Table 1. Users of ACEI/ARB were found to be significantly older than non-ACEI/ARB users (54.4 vs. 40.5 years), were more often male (70.5 vs. 29.5%) and non-Kuwaiti (40.3 vs. 59.7%). Additionally, ACEI/ARB users were found to have more co-morbidities, for instance; diabetes (45.8 vs. 14.8%) and hypertension (92.9 vs. 13.0%). The proportion of patients on ACEI/ARB admitted into the ICU were proportionally more than non-ACEI/ARB users (11.1 vs. 7.6%). Even more, mortality was greater among ACEI/ARB users (6.5%) when compared to non-ACEI/ARB users (4.3%).

**Table 1:** Baseline characteristics of COVID-19 patients by ACE/ARB and non-ACE/ARB use.

	**ACE/ARB users**		**Non-ACE/ARB users**		
	***n*** **= 325 (8.1%)**		***n*** **= 3694 (91.9%)**		***P*-value**
Age, y	54.4	(10.2)	40.5	(16.8)	<0.001
<18 years	1	(0.3)	293	(7.9)	
18-29	5	(1.5)	660	(17.9)	
30-39	19	(5.8)	974	(26.4)	
40-49 years	85	(26.2)	725	(19.7)	
50-59 years	115	(35.4)	540	(14.6)	
60 years and above	100	(30.8)	495	(13.4)	
Gender					
Female	96	(29.5)	1093	(29.5)	0.99
Male	229	(70.5)	2601	(70.4)	
Nationality					
Kuwaiti	194	(59.7)	2175	(58.9)	0.78
Non-Kuwaiti	131	(40.3)	1519	(41.1)	
Systolic Blood Pressure, mmHg	137.1	(18.3)	125.1	(16.1)	<0.001
Diastolic Blood Pressure, mmHg	81.7	(10.6)	77.1	(9.8)	<0.001
Heart Rate, bpm	88.1	(14.2)	88.2	(15.0)	0.92
Diabetes, *n* (%)	149	(45.8)	585	(14.8)	<0.001
Hypertension, *n* (%)	302	(92.9)	480	(13.0)	<0.001
Coronary artery disease, *n* (%)	36	(11.1)	132	(3.6)	<0.001
Chronic Obstructive Pulmonary Disease, *n* (%)	4	(1.2)	13	(0.4)	0.02
Asthma, *n* (%)	25	(7.7)	212	(5.7)	0.15
Temperature on admission	37.0	(0.6)	36.9	(0.6)	0.05
ICU admission, *n* (%)	36	(11.1)	282	(7.6)	0.03
Mortality, *n* (%)	21	(6.5)	160	(4.3)	0.76

Statistical significance set at p < 0.05.

*ICU; Intensive Care Unit*.

Table 2 shows the unadjusted and adjusted odds ratios from the logistic regression analysis. ACEI/ARB use was found to be significantly associated with greater odds of ICU admission compared to non-users in the unadjusted analysis [OR, 1.51 (95% CI: 1.04–2.19), *p* = 0.028]. However, after adjustment for confounding factors (age, gender, non-Kuwaiti, coronary artery disease, diabetes, and hypertension), ICU admission was found to be inversely associated with ACEI use [OR, 0.57 (95% CI: 0.34–0.88), *p* = 0.01]. Following the adjustment for confounding factors, ACEI/ARBs use was found to be inversely associated with mortality [OR, 0.56 (95% CI: 0.33–0.95), *p* = 0.032].

**Table 2:** Odds ratios for ACE/ARB use vs. Non-ACE/ARB use with ICU admission and mortality.

	**Unadjusted model**		***P* value**	**Fully adjusted model**		***P* value**
	**OR**	**(95% CI)**		**OR**	**(95% CI)**	
Mortality	1.52	(0.95–2.44)	0.078	0.56	(0.33–0.95)	0.032
ICU Admission	1.51	(1.04–2.18)	0.028	0.57	(0.34–0.88)	0.010

Fully adjusted model includes the following covariates: age, gender, non-Kuwaiti, CAD, diabetes, hypertension.

*Statistical significance set at p < 0.05*.

Table 3 shows the adjusted analysis restricted to patients aged over 40 years old with or without coronary artery disease (CAD). For patients over 40 years old, logistic regression analysis showed that ICU admission was inversely associated with ACEI/ARB use [OR, 0.51 (95% CI: 0.33–0.78), *p* = 0.002]. This association was also observed when analysis was restricted to patients >40 years with a history of CAD [OR, 0.54 (95% CI: 0.35–0.84), *p* = 0.006]. Mortality was also inversely associated with ACEI/ARB use, for those over 40 years [OR, 0.47 (95% CI: 0.27–0.80), *p* = 0.005] and >40 years with a history of CAD (OR, 0.44 [95% CI: 0.25–0.78), *p* = 0.005].

**Table 3:** Odds ratios for ACE/ARB use vs. non-ACE/ARB use with ICU admission and Mortality for patients over 40 years.

	**Fully adjusted model**		***P* value**
	**OR**	**(95% CI)**	
Mortality^a^	0.47	(0.27–0.80)	0.005
Mortality^b^	0.44	(0.25–0.78)	0.005
ICU Admission^a^	0.51	(0.33–078)	0.002
ICU Admission^b^	0.54	(0.35–0.84)	0.006

Fully adjusted model includes the following covariates: age, gender, non-Kuwaiti, CAD, diabetes, hypertension.

a Restricted to patients over 40 years old (n = 2,067).

b Restricted to patients over 40 years old with history of cardiovascular disease (n = 775).

*Statistical significance set at p < 0.05*.

## Discussion

Among COVID-19 positive patients, the present study found a significant inverse association between ACEI/ARBs use, ICU admission, and mortality following the adjustment for baseline demographics and co-morbidities. Several studies have postulated that the use of ACEIs/ARBs may influence COVID-19 severity (18–20). However, the mechanism by which these drugs affect the pathogenesis of COVID-19 disease remains unclear, and there is need for clinical studies to guide the usage of such drugs in patients with COVID-19 disease (21).

SARS-CoV-2 uses angiotensin-converting enzyme 2 (ACE2) receptor to reach and replicate in the mucosal epithelium of the respiratory tract (8). Because previous research on animal studies (22, 23) have shown that the use of ACEI/ARBs can upregulate ACE2 receptor expression (13), concerns were raised on the subsequent effect of this on COVID-19 disease propagation (24). It was postulated that the use of such drugs may increase patient susceptibility of acquiring COVID-19 disease and/or having a more severe clinical course (24) and thus requiring the discontinuation of these drugs in suspected cases. However, the findings from animal studies remain equivocal, and the consequences of ACE2 receptor upregulation requires further investigation. Conversely, some studies have indicated that ACE 2 receptor upregulation may initiate an anti-inflammatory state by augmenting vasodilatation and providing antioxidant protective effects (21, 25, 26). These effects may be enhanced through a mechanism by which an increase in Angiotensin I (Ang 1–7) production exerts anti-inflammatory and antioxidant properties once bound to its receptor (26). The protective anti-inflammatory effects of ACE2 and Ang 1–7 were evident in studies conducted on animal lung injury models (21, 27), and those involving cardiac myocytes, in part due to their role in regulating cardiac contractility and hypoxia-induced cardiac genes (28).

The continued use of ACEI/ARBs for patients diagnosed with COVID-19 disease was initially concerning because a few studies reported it to be associated with worse outcomes (18–20). This was a result of their crude analysis showing these drugs to be associated with increased COVID-19 disease severity. In fact, the effect of demographics and co-morbidities on disease severity in COVID-19 is well established. For example, the overall mortality of COVID-19 is reported to be 1–5%, but when stratified by age, it can go as high as 14.8% for those who are over 80-years. In addition, in a cohort of 44,672 confirmed cases, the case-fatality rate of patients with COVID-19 disease who have co-morbidities was found to be higher than average, these include cardiovascular disease (10.5%), diabetes (7.3%), chronic respiratory disease (6.3%), and hypertension (6%) (8, 9). For this reason, when reporting associations relating to COVID-19 disease, demographics, and co-morbidity status has to be taken into consideration.

Although a retrospective study on 1,178 hospitalized COVID-19 disease patients in Wuhan City found that the use of ACEIs/ARBs was not associated with COVID-19 disease severity or mortality, the study did not adjust for confounding variables (29). Similarly, Tetlow et al. (30) reported that ACEI/ARB use was not associated with acute kidney injury, macrovascular thrombi, or mortality when studying 558 hospital inpatients admitted with COVID-19 disease. Moreover, an observational study by Braude et al. (31) on 1,371 patients from 11 hospitals in the United Kingdom, found that although ACEI/ARB use was not associated with increased inpatient mortality, their use was found to be associated with shorter length of in-hospital stay, in particular the effect was stronger in hypertensive patients.

After adjusting for confounding variables, the potential beneficial effect of ACEI/ARBs use becomes more evident (24). For instance, Zhang et al. (32) reported a lower mortality risk in the ACEI/ARB group as compared to non-ACEI/ARB group after adjusting for age, gender, comorbidities, and in-hospital medications [HR, 0.42 (95% CI, 0.19-0.92); *P* = 0.03] but the study population was limited to hypertensive patients, which in turn limits generalizability. Fosbol et al. (24), on the other hand, conducted a study on 4,480 patients with COVID-19 disease, and reported that ACEI/ARB was not found to be associated with increased COVID-19 disease severity after adjusting for age and co-morbidities [HR, 1.15 (95% CI 0.95–1.41)]. The results of our study replicate this finding and also reported ACEI/ARBs to be protective against severe COVID-19 disease, with decreased need for ICU admission [OR, 0.57 (95% CI: 0.34–0.88), *p* = 0.01]. Fosbol et al. (24) also reported their findings on the association of ACEI with COVID-19 disease-related mortality. Although ACEI/ARBs were found to be significantly associated with mortality in unadjusted analysis [HR, 2.65 (95% CI 2.18–3.23)], this association was lost when adjusted for age and medical co-morbidities [HR, 0.83 (95% CI 0.67–1.03)]. Our study was able to replicate this finding with a greater effect size as our adjusted OR for mortality was 0.56 and can be as low as 0.33 when comparing patients with COVID-19 disease using ACEI/ARBs to those who do not use ACEI/ARBs. As presented in our regression model, we have adjusted for nationality (Kuwaiti vs. Non-Kuwaiti) as a confounding variable based on the findings of our previous study, which found Non-Kuwaitis to have a two-fold higher odds of death or ICU admission, which is explained by the differences in socioeconomic status, living and working conditions, and health care access between the two groups (33).

Similarly, a retrospective study by Senkal et al. (34) on 611 COVID-19 patients in Istanbul found that a total of 165 patients had severe disease (hospitalization for >14 days, ICU admission, or death), and the use ACEI was found to be significantly associated with lower disease severity [OR, 0.37 (95% CI 0.15–0.87)], milder infiltrations on CT, lower level of inflammatory markers (C-reactive protein and ferritin), and shorter hospital stay. Moreover, although their study also found ARB exposure to be associated with lower odds of severe disease, this association failed to reach significance [OR, 0.6 (95% CI 0.27–1.36) *p* = 0.31].

Comparable to previous findings, Zhou et al. (35) assessed 15,504 patients from 17 different hospitals in China to investigate the association between in hospital use of ACEI/ARB with 28-day all cause death of COVID-19 in 3,572 patients. The authors reported the results of their propensity score-matched analysis, in which patients were matched for age, gender, disease severity, co-morbidities, and calcium channel blocker usage after adjustment for imbalanced variables and in-hospital medications. They found that in-hospital ACEI/ARB use was associated with decreased risk of 28-day all-cause mortality from COVID-19 in patients with hypertension [OR, 0.11 (95% CI 0.15–0.66), *p* = 0.002], hypertension and coronary artery disease (CAD) [OR, 0.11 (95% CI 0.04–0.31) *p* < 0.001], and CAD [OR, 0.38 (95% CI 0.16–0.89) *p* = 0.03].

More recently, similar to the present study, Bean et al. (36) evaluated the risk of COVID-19 disease severity among 1,200 patients. A total of 33% (*n* = 399) of the patients were found to be using ACEI or ARB and the adjusted risk of ICU admission or death was lower among users [OR, 0.63 (95% CI 0.47–0.84)].

Comparatively, the beneficial effects of ACE use have been reported in heterogenous patient cohorts from different demographic backgrounds. A more recent meta-analysis of 12 studies, including 19,000 COVID-19 positive patients found that ACEI/ARB use did not increase the risk of disease severity (OR = 0.98; 95 % CI, 0.87–1.09; *p* = 0.69) or mortality (OR = 0.73, 95 %CI, 0.5–1.07; *p* = 0.111) in patients with COVID-19, and the use of ACEI/ARB was found to protective against mortality among hypertensive patients when compared with other antihypertension medications [OR = 0.48, (95 % CI, 0.29–0.81); *p* = 0.006] (37). Another meta-analysis by Flacco et al. (38) combined the results of 10 studies with a total of 9,890 patients with hypertension to assess if ACEI/ARB use was associated with severe or lethal COVID-19 disease and found no significant association with either ACEI [OR: 0.9 (95% CI 0.65–1.26)] or ARB [OR: 0.92 (95% CI 0.75–1.12)] use. The available findings in the literature in conjunction with the findings of these studies supports the statements made by professional societies about continuation of ACEI/ARBs for patients with COVID-19 disease (39).

Although the present study did not assess the relationship between ACEI/ARB use and COVID-19 incidence, it was postulated that the use of ACEI/ARBs may potentially increase the risk of acquiring COVID-19 disease. However, evidence is limited and remains equivocal. For example, in one study by Reynolds et al. (40) that included 12,594 COVID-19 positive found no association with any medication class, which included ACEIs/ARBs, calcium channel blockers, beta blockers, and thiazide diuretics. Reynolds et al. (40) also reported that none of the examined drug classes were associated with an increased severity of COVID-19 disease (40).

The association of ACEI and ARBs with COVID-19 disease is complex and further studies are needed to explore the mechanism of interaction of SARS-CoV-2 virus with ACE2 receptor and its implications on COVID-19 disease pathophysiology. Bellone and Calvisi (41) have discussed the association of specific ACE2 polymorphisms on the aggressiveness of COVID-19 disease and suggested that Ins/Del and Del/Del polymorphisms may be associated with severe clinical disease and mortality from Acute Respiratory Distress Syndrome (ARDS). It was also proposed that ACE and Angiotensin II may be a therapeutic target for COVID-19 patients through their effects on the previously discussed polymorphisms (41). Recently, Vaduganathan et al. (21) reviewed the involvement of the RAAS system in COVID-19 disease pathophysiology and indicated the benefits of ACEI/ARB use to outweigh the hypothesized risks of these medication classes on COVID-19 disease incidence or severity among patients, in otherwise stable condition, with indications to take these drugs. Altogether, the current evidence in the literature is in conjunction with the findings of this study and supports the statements made by professional societies about continuation of ACEI/ARBs for patients with COVID-19 disease (39, 42).

## Limitations

The present study has several limitations. Firstly, the observational nature of the study does not confer causality but rather our results are reported as associations. Secondly, this study was limited to a single hospital in Kuwait so generalizability could be affected. Although the medication list for all patients is accurately reported in the system, indication for ACEI/ARB use was not gathered for all patients. Those not on ARB/ACE may be on an alternate treatment or on no treatment, which could result in some residual confounding. We also did not collect data on medication dose, duration of therapy, and have not investigated differences between the use of ACEI or ARB, which add up to the study's limitations.

## Conclusion

The current evidence in the literature supports continuation of ACEI/ARB medications for patients with co-morbidities that acquire COVID-19 infection. With time, the protective effects of such medications on COVID-19 disease severity and mortality is becoming clearer, and the findings of the present study support the use of ACEI/ARB medication in such patients.

## Data Availability Statement

The raw data supporting the conclusions of this article will be made available by the authors, without undue reservation.

## Ethics Statement

Ethical approval for conduction of this study was granted by the Ministry of Health Ethical Review Board in Kuwait. All participants provided written informed consent.

## Author Contributions

SAlS: conception of idea, overseeing project, and proofreading. RE and DA: collecting data and writing. SAlY: analyzing data and writing of paper. HB: analyzing data and writing. SAlm: analyzing data, writing, and proofreading. MA-H and MJ: overseeing project and proofreading. All authors contributed to the article and approved the submitted version.

## Conflict of Interest

The authors declare that the research was conducted in the absence of any commercial or financial relationships that could be construed as a potential conflict of interest.
